# Educating physicians in evidence based medicine: current practices and curricular strategies

**DOI:** 10.1007/s40037-016-0301-5

**Published:** 2016-10-25

**Authors:** Lauren A. Maggio

**Affiliations:** Department of Medicine, Uniformed Services University of the Health Sciences, Bethesda, MD USA

**Keywords:** Evidence-based medicine, Information storage and retrieval, Curriculum, Undergraduate medical education

## Abstract

**Introduction:**

Evidence-based medicine (EBM) is an expectation of professional healthcare and a requisite component of medical school curricula. However, upon graduation medical students’ EBM skills have been found lacking suggesting a need to examine EBM training.

**Methods:**

This PhD report presents two studies on EBM education. The first study is a literature review that describes and attempts to assess educational interventions for teaching medical students EBM. The second study presents a multi-institutional case study conducted in North America using interviews and curricular materials to identify EBM instructors’ perceptions of challenges that may impede medical students’ efforts to learn EBM.

**Results:**

The literature review analyzed 20 learning interventions from 12 countries that were presented in classrooms (75 %) and clinics (25 %). The steps of EBM were addressed to varying degrees. It was not possible to draw conclusions about the efficacy of the interventions due to lack of detailed reporting. The qualitative study identified four learning challenges: sub-optimal role models, student lack of willingness to admit uncertainty, lack of clinical context, and difficulty mastering EBM skills. To meet these challenges, participants described interventions such as integrating EBM skills with other content/courses, incorporating clinical content into EBM teaching, providing faculty development, using whole-task EBM activities, and longitudinal integration of EBM across the curriculum.

**Conclusion:**

This PhD report takes steps to describe and assess EBM learning interventions, presents student learning challenges and looks at approaches institutions take to meet them. Educators can use these findings to examine their curriculum and learning environments and, if desired, adopt them for their training.

## Introduction

The practice of evidence-based medicine (EBM) ideally combines available research evidence, physician expertise, and the patient’s wishes [1]. The steps of EBM include clinicians recognizing a gap in their knowledge, articulating the gap as a clinical question, acquiring and appraising relevant evidence, applying the evidence to the patient’s care, and evaluating their practice in light of the evidence [2]. Since its introduction over 20 years ago, EBM has become the gold standard of clinical practice [3] and key component of most medical schools’ curricula [4].

Despite curricular integration of EBM, researchers have found clerkship-level medical students able to execute only half of the steps of EBM with difficulties especially in critically appraising the evidence found [5]. This deficiency suggests a need for future research to focus on the effectiveness of EBM interventions and educational approaches designed to overcome these challenges. Therefore, we performed four studies to explore how EBM is practised and taught to medical students. This PhD report focuses on two of these studies that describe educational interventions for learning EBM and EBM instructors’ perceptions of challenges that may impede medical students’ efforts to learn EBM.

## Methods

We reviewed the literature on educational interventions to teach medical students EBM skills published between 2006–2011 by searching MEDLINE, Scopus, Evidence-Based Medicine Reviews, and ERIC using relevant medical subject headings and keywords [6]. Articles were excluded that did not include medical students, focused on a single EBM skill, or only examined existing EBM knowledge, skills, or attitudes. Relevant full-text articles were reviewed and data extracted to characterize educational settings, EBM skills covered, teaching methods used, learners and instructors. Using the Best Evidence in Medical Education collaboration’s modified four-level Kirkpatrick’s hierarchy, we attempted to evaluate the impact of the interventions on 1) learners’ views of the intervention, 2a) learners’ attitudes towards the intervention, 2b) learners’ modification of EBM knowledge or skills, 3) learners’ EBM behaviour changes, changes in organizational practice, or 4) benefits to patient care [7]. We utilized Khan’s hierarchy of EBM teaching and learning methods to rate learning activities based on its three levels. Level 1 activities are interactive, such as case discussions or role play scenarios, and clinically integrated in that they include clinical content or are implemented in clinical settings. Level 2(a) activities are interactive, but classroom-based. Level 2(b) includes didactic (lecture-based) but clinically integrated activities. Level 3, includes didactic, classroom-based activities [8].

We next conducted a multi-institutional case study to identify challenges that may impede students in learning EBM and the educational approaches employed to overcome them [9]. Using a semi-structured interview protocol, we interviewed 31 EBM instructors (17 clinicians, 11 librarians, 2 educationalists, and 1 epidemiologist) from 17 medical schools (13 in the United States; 4 in Canada) that were identified as graduating students confident in their EBM abilities based on the Association of American Medical Colleges Graduation Questionnaire (GQ). The GQ represents self-reported data from graduating medical students in North America related to their medical school experience. We elected to interview instructors instead of students because we felt they would be most knowledgeable about their institution’s EBM curriculum. Additionally, we believed that EBM instructors over time would have encountered multiple medical students and could speak of their varied student experiences with challenges and the institution’s curricular attempts to meet them. Thirteen participating institutions also supplied curricular materials for review. We inductively analyzed the interviews and created profiles of each institution’s EBM training. Based on these profiles, we identified, through rounds of discussion, EBM learning challenges and educational approaches used by the institutions to meet them.

## Results

Our literature review identified 20 educational interventions from 12 countries that were presented in classrooms (75 %) and clinics (25 %). Twenty percent of interventions also included online components. Interventions addressed the steps of EBM to varying degrees (Table 1). We were unable to draw conclusions about the efficacy of interventions using Kirkpatrick’s hierarchy [8] due to lack of detailed reporting. Based on Khan’s three-level hierarchy, we determined that 40 % were level 1 (interactive and clinically integrated), 40 % were level 2a (interactive, but classroom based) or 2b (didactic, but clinically integrated), and 20 % level 3 (didactic, classroom-based).

**Table 1:** Coverage of evidence-based medicine steps in educational interventions

EBM Steps	Number of interventions including the step
Step 1: Recognizing a knowledge gap	4 (20 %)
Step 2: Articulating a clinical question	18 (90 %)
Step 3: Acquiring information	18 (90 %)
Step 4: Appraising information	17 (85 %)
Step 5: Applying evidence to patient care	13 (65 %)
Step 6: Evaluating practice	1 (5 %)

By analyzing EBM instructor interviews and curricular materials, we identified instructors’ perceptions of four student challenges or factors that may impede students’ ability to learn EBM: sub-optimal role models, student lack of willingness to admit uncertainty, lack of clinical context, and difficulty mastering EBM skills. The observation of sub-optimal role models was described with particular concern. Participants feared sub-optimal role models might discourage EBM practice by exhibiting poor attitudes towards EBM, demonstrating weak EBM skills, and failing to make explicit their use of EBM in clinical practice. Participants reported several approaches to overcome these challenges. Five of the approaches were most commonly applied, namely integrating EBM skills with other content/courses, incorporating clinical content into EBM teaching, providing faculty development, using whole-task EBM activities, and longitudinal integration of EBM across the curriculum. For example, institutions described faculty development efforts that trained instructors to be cognizant of their status as EBM role models and vocalize their uncertainty in conjunction with walking students through their execution of all EBM steps.

## Discussion

Based on a review of the literature, this PhD report aims to describe how EBM is taught and to assess the effectiveness of the teaching approaches. Additionally, based on interviews with North American EBM instructors, it identifies challenges that impede students in learning EBM and potential approaches to overcoming them. Unfortunately, in our literature review, due to lack of reporting in the reviewed articles, we were unable to draw conclusions as to the efficacy of the interventions, which aligns with recent systematic reviews of EBM education [10, 11]. This lack of detail is a weakness, which hopefully will be remedied by recently published guidelines for reporting EBM educational interventions [12].

Based on our findings and experiences as EBM instructors and practitioners, we propose several recommendations for modifying EBM training. For example, we noted that the first (recognizing a knowledge gap) and last (evaluating practice) EBM steps are not well covered. Research suggesting that students struggle with critical appraisal skills [5] may indicate that these skills are more difficult for students to master and may explain why less attention is paid to the first and last steps. However, based on our finding that students lack willingness to admit uncertainty, which directly impacts their ability to recognize knowledge gaps, and medicine’s emphasis on continuous improvement we recommend EBM training take a more comprehensive approach to include all EBM steps. Future research should investigate the difficulty levels of all EBM steps to inform instructors in their approach to training. Additionally, most interventions targeted clinical-level students. However, research indicates that introducing preclinical students to EBM raises learners’ self-efficacy in practising EBM and the likelihood that they will continue to practise EBM [13]. Therefore, we suggest that EBM training be introduced in preclinical training. As a literature review, this study has limitations, including only capturing published interventions. It is possible institutions provide students robust EBM training, but their efforts are unpublished.

Our interviews with EBM instructors indicated that while the students at their institutions reported confidence in their EBM abilities, instructors felt that the challenges identified hindered students in learning EBM. The identification of these learning challenges adds to the EBM literature, which has previously focused on describing the barriers residents face when practising EBM [14]. These findings provide educators a window into the student experience of learning EBM and may inform future training. Related to the challenges, we identified five approaches to overcome them as described by the participating institutions. Although we have no empirical proof of their efficacy for EBM training or overcoming these challenges, we encourage EBM instructors to consider them in their design of EBM training, as they are consistent with learning theory. For example, the suggestion to integrate EBM training into clinical settings aligns with situated learning theory that encourages that learning be set in the context of the culture and place in which it naturally occurs [15].

While this study provides new insights into student learning challenges and educational approaches, it has limitations. Although participants described their approaches to meet the identified student challenges it was not possible for us to determine if they would be necessarily appropriate for other institutions. Additionally, we conducted interviews with only North American participants. While our findings may be relevant to EBM educators outside of North America, future researchers might seek to interview a more global sample of EBM educators to confirm and expand the identified challenges and approaches.

## Conclusion

In this PhD report, we have described EBM learning interventions for medical students and noted an imbalance in the coverage of EBM steps, such that the first (recognizing a knowledge gap) and last (evaluating practice) steps are less well covered. Based on our finding that students lack willingness to admit uncertainty, which impacts their ability to recognize knowledge gaps, and medicine’s emphasis on continuous improvement, we recommend EBM training take a more comprehensive approach to include all the EBM steps. Based on interviews, we also identified student learning challenges, including a notable concern about suboptimal faculty role models, and the approaches that institutions take to meet them, such as faculty development. We hope our findings will inform EBM educators and inspire future EBM research.

## Advice

Completing a PhD can be long and lonely endeavour. Early in the process find a writing partner that you can meet in-person or check-in with online. Your writing partner will help you stay focused and productive.
